# Maternal LPS Exposure Enhances the 5-HT Level in the Prefrontal Cortex of Autism-like Young Offspring

**DOI:** 10.3390/brainsci13060958

**Published:** 2023-06-15

**Authors:** Fang Lin, Xinyuan Wang, Ruifang Luo, Binlin Yuan, Shasha Ye, Ting Yang, Lu Xiao, Jie Chen

**Affiliations:** 1 Chongqing Key Laboratory of Childhood Nutrition and Health, Children’s Nutrition Research Center, Children’s Hospital of Chongqing Medical University, Chongqing 400015, China; linfangdoctor@163.com (F.L.); m13500313975@163.com (L.X.); 2 Ministry of Education Key Laboratory of Child Development and Disorders, National Clinical Research Center for Child Health and Disorders, Chongqing 400015, China; 3 Department of Gastroenterology, Children’s Hospital of Chongqing Medical University, Chongqing 400015, China

**Keywords:** serotonin (5-HT), tryptophan metabolism, medial prefrontal cortex (mPFC), autism spectrum disorder (ASD), lipopolysaccharide (LPS)

## Abstract

Autism spectrum disorder (ASD) is a neurodevelopmental disorder characterized by reduced social interactions, impaired communication, and stereotyped behavior. The aim of this research is to investigate the changes in serotonin (5-HT) in the medial prefrontal cortex (PFC) of autism-like offspring induced by maternal lipopolysaccharide (LPS) exposure. Pregnant Sprague-Dawley rats were intraperitoneally injected with LPS to establish an autism-like model in their offspring. Offspring prenatally exposed to LPS showed autism-like behavior. The serotonin level in the mPFC of 2-week-old offspring was noticeably increased after maternal LPS exposure. Differentially expressed genes (DEGs) were enriched in pathways related to tryptophan metabolism and the serotonin system, as shown in RNA-seq findings. Consistently, tryptophan and serotonin metabolisms were altered in 2-week-old LPS-exposed offspring. The mRNA expression levels of 5-HT catabolic enzymes were remarkably reduced or tended to decrease. Moreover, maternal LPS exposure resulted in a higher serotonin 1B receptor (5-HT_1B_R) expression level in the mPFC but no difference in tryptophan hydroxylase 2 (TPH2) or serotonin reuptake transporter (SERT). The concentrations of 5-HT in serum and colon were increased in LPS-exposed offspring. Meanwhile, the expression level of tryptophan hydroxylase 1 (TPH1) in the colon was increased after maternal LPS treatment, whereas SERT was reduced. Furthermore, Golgi-Cox staining showed that neuronal dendritic length and spine density were significantly reduced in the mPFC of LPS-exposed offspring. The current study reveals that maternal LPS treatment resulted in an exaltation of the 5-HT of mPFC in ASD-like young rats, which may partly be caused by the abnormal elevation of 5-HT metabolism in its colon.

## 1. Introduction

Autism spectrum disorder (ASD) is a group of neurodevelopmental disorders characterized by various behavioral symptoms such as social deficits, repetitive behaviors, and anxiety [1]. To a great extent, the etiopathogenesis of ASD is multifactorial, as it is caused by both genetic and environmental factors and their interactions [2,3]. In fact, numerous studies have found an association between maternal infection and ASD [4,5]. Lipopolysaccharide (LPS), an endotoxin from the cell wall of Gram-negative bacteria, can imitate bacterial infection during pregnancy and induce sickness behavior in offspring [6].

As a key neurotransmitter, 5-HT regulates multiple aspects of neural development [7,8]. Abnormal serotonergic neurotransmission may be important to the pathogenesis of autism. Serotonin synthesis has been found to be asymmetric in the dentatothalamocortical pathway in autistic boys, with unilateral decreases in serotonin synthesis in the frontal cortex and thalamus and increased serotonin synthesis in the contralateral dentate nucleus of the cerebellum [9]. The changes in 5-HT in the brain have also been reported on animal experiments. As a putative animal model for autism, maternal valproic acid exposure resulted in increases in serotonin in the hippocampal and prefrontal cortex [10,11]. In addition, reductions in serotonin tissue content and the density of serotonin axons in the hippocampus have been found in the inbred mouse strain BTBR T+ Itpr3tf/J, accompanied by possible compensatory changes in serotonin neurons [12]. Previous articles published by our research group show that serum 5-HT levels were significantly higher in children with ASD compared to control children, which were closely related to the symptom severity of children with autism [13]. Our recent study also found that the metabolic disturbances of ASD children involved in 5-HT-related pathways and differential gut metabolites were correlated with autistic symptoms and neurodevelopmental levels [14]. It is well known that ASD is a neurodevelopmental disorder, so our aim is to explore whether there are changes in 5-HT levels in the prefrontal cortex at the early stage of neurodevelopment period.

To investigate this hypothesis, the rats were first treated with LPS during pregnancy to establish an ASD-like offspring model, and the 5-HT level of the mPFC was measured. Then, we combined RNA-seq techniques and tryptophan target metabolomics to identify abnormalities in 5-HT metabolism-related signaling pathways of the mPFC in the offspring. Finally, the expression levels of metabolic enzymes and 5-HTRs were confirmed in both the mPFC and colon of LPS-exposed offspring. The current study offers an experimental foundation for the possible mechanism of 5-HT alterations during the early neurodevelopment period.

## 2. Materials and Methods

### 2.1. Animals and Drug Treatment

Sprague-Dawley (SD) rats were acquired from the Animal Experiment Centre of Chongqing Medical University (Chongqing, China) and raised in a specific pathogen-free environment with a 12 h light-dark cycle (light: 7 a.m.–7 p.m.; dark: 7 p.m.–7 a.m.) at a constant temperature (22–24 °C). The animal use protocol has been reviewed and approved by the Ethics Committee of the Children’s Hospital of Chongqing Medical University (CHCMU-IACUC20200424016, CHCMU-IACUC20220629014).

**Figure 1:**
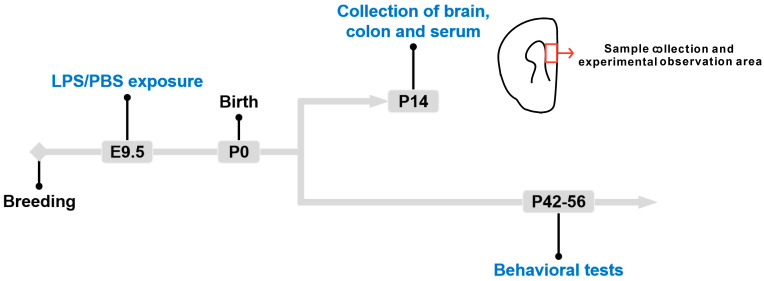
Schematic diagram of the PBS and LPS groups. LPS offspring (*n* = 40 from 6 litters of rats) received a single dose of 100 µg /kg LPS by intraperitoneal injection at 9.5 days of gestation, and the PBS rats (*n* = 40 from 6 litters of rats) received the same volume of PBS. The day of birth was PND 0. The brain, colon, and serum of offspring (*n* = 20 per group from 3 litters of rats) were collected on PND 14 for the tests (enzyme-linked immunosorbent assay, immunofluorescence staining, RNA sequencing analysis, tryptophan target metabolomics, polymerase chain reaction (PCR), Western blot (WB), and Golgi staining). The behavioral tests were conducted from PND 42 to PND 56 on other offspring (*n* = 20 per group from 3 litters of rats).

Eight-week-old female rats were paired with two male rats of the same strain and maintained overnight. Then, female rats with a vaginal plug were observed the next morning. These females were observed at 0.5 days of gestation. Meanwhile, they were randomized to the PBS and LPS groups. LPS (Sigma, St. Louis, MO, USA) was dissolved at 50 µg/mL in phosphate-buffered solution (PBS) and administered intraperitoneally to the pregnant rats in the LPS group on 9.5 days of gestation (100 µg /kg) (Figure 1). Similarly, the rats in the PBS group received an equal volume of PBS at the same gestational timepoint. The male offspring used for further assays were classified as LPS (*n* = 40 from 6 litters of rats) and PBS (*n* = 40 from 6 litters of rats) groups, respectively.

### 2.2. Behavioral Tests

All behavioral tests were performed with 6- to 8-week-old rats (*n* = 20 per group) during the day (light-on period). The tests were conducted in a peaceful and mild environment to ensure the quality of the experiment. Before the next set of rats were tested, the test boxes and cages were cleaned of urine and feces and then disinfected with 75% alcohol. 

### 2.3. Open-Field Test

The open-field test was conducted according to a previous methodology [15]. The rats were acclimated in the testing room and left alone for 30 min before the test. A square box (40 cm [length] × 40 cm [width] × 30 cm [high]) was used to evaluate the exploratory activity of rats. The floor area included both the surrounding and center regions. At the beginning of the trial, each rat was placed in the middle zone. The ANY-Maze Video Tracking System (ANY-Maze, St. Louis, MO, USA) tracked the rat’s movements for 5 min. Moreover, the total traveled distance and the number of lines crossing for each animal were recorded using the software. The grooming time was recorded independently by two experimenters. Both of them were blinded to the group assignments.

### 2.4. Three-Chamber Sociability Test

The three-chamber sociability test was conducted in a white plexiglass box (60 × 40 cm^2^) with three chambers (20 × 40 cm^2^) and entrances (6 × 6 cm), allowing access to the side chambers. The test consisted of two 5 min stages: the social interaction test and the social novelty test. The social interaction test involves an age- and sex-matched stranger rat and a tiny item in the two side chambers [16]. The social novelty test includes a stranger rat and a familiar rat in the two side chambers [16]. On the day before the test, the rats were habituated to the apparatus for 5 min, with an empty cage in each of the two side chambers. The rats were placed in the central chamber and permitted to explore the cage freely at each stage of the test. The ANY-Maze Video Tracking System was used for recording the duration in each chamber. Between trials, the device was cleaned.

### 2.5. Animal Tissue Collection

Male offspring aged 2 weeks were sacrificed using phenobarbital sodium. Following the macrocutting of the brain, the medial prefrontal cortex (mPFC) was first quickly frozen in liquid nitrogen and then frozen for long-term preservation at −80 °C. Meanwhile, the serum and colon of each subject were extracted and stored at −80 °C. The tissue collection was completed by two experiments that were blinded to the group assignments.

### 2.6. Enzyme-Linked Immunosorbent Assay

Residual blood from the mPFC (*n* = 10 per group) and colon (*n* = 8 for PBS, *n* = 12 for LPS) was rinsed away, and the tissue was homogenized in 1× PBS (100 mg tissue/1 mL PBS). The tissue was ground into pieces, and the homogenates were centrifuged at 5000× *g* for 5 min at 4 ℃. The supernatant (*n* = 10 for PBS, *n* = 14 for LPS) was collected, and the 5-HT concentration was determined using an ELISA kit (Elabscience, 5-HT, Wuhan, China), as directed by the manufacturer. The absorbance was measured at 450 nm, and the optical density values were computed in triplicate using a standard curve.

### 2.7. Immunofluorescence Staining

The rats (*n* = 4 per group) were perfused transcardially and sacrificed at 2 weeks of age, and their brains were totally exfoliated and preserved in 4% paraformaldehyde. Then, the brain was dehydrated with 4% paraformaldehyde containing 15% and 30% sucrose in that order. The brain was then sectioned into 15 μm thick sagittal slices by using a cryostat (Leica, Wetzlar, Germany) after the tissue had sunk to the bottom. Then, 0.3% Triton X-100 was applied to the frozen slices for 20 min before blocking them with 5% BSA (Solarbio, Beijing, China) for 1 h at room temperature. The slices were then incubated at 4 °C overnight with primary antibodies, including 5-HT (GTX31099, Genetex, SC, USA) and 5-HT_1B_R (DF3497, Affinity, Golden, CO, USA). Fluorescent secondary antibodies, including donkey anti-rabbit (ab150074, Abcam, Cambridge, UK) and donkey anti-mouse (ab150105, Abcam, Cambridge, UK), were applied at room temperature in the dark for 1 h. Nuclei were labeled with DAPI solution (Sigma, St. Louis, MO, USA). Finally, fluorescent images of the mPFC were captured using a Nikon automated bioluminescence microscope at a magnification of 400×. A single color channel analysis was performed using Image J software (version 1.8.0) to quantify the mean gray value. Mean gray value = Integrated Density/Area.

### 2.8. Golgi-Cox Staining

Golgi-con staining was performed using the Hito Golgi-Cox OptimStainTM Kit (Hitobiotec, Kingsport, TN, USA) One day before staining, solution 1 and solution 2 were mixed evenly in a 1:1 ratio and stored at room temperature in the dark. After careful extraction and washing in PBS, the brain (*n* = 4 per group) was immersed in a 10 mL mixed solution and stored at room temperature in the dark for 2 weeks. Two weeks later, the tissue was transplanted into the same volume of solution 3 and maintained for 3 days at 4 °C. The brain was cut at the coronal plane (200 µm thick), and the Hito Golgi-Cox OptimStain PreKit (Hitobiotec, Kingsport, TN, USA) was used for section staining. ImageJ software and NeuronStudio software (version 0.9.92) were used for analyzing the dendritic length and spine density, respectively.

### 2.9. Quantitative Polymerase Chain Reaction

As instructed by the manufacturer, the Total RNA Extraction Kit (Promega Biotech, Beijing, China) was used for RNA extraction from the samples. The RNA concentration was measured using the NanoDrop ONE System (Thermo Scientific, Waltham, MA, USA). The PrimeScript RT Reagent Kit (Takara, Kyoto, Japan) was used for cDNA synthesis. The quantitative polymerase chain reaction was performed using SYBR Green Mix (Takara, Kyoto, Japan) on a real-time PCR system (Bio-Rad, Hercules, CA, USA) for 40 cycles (95 °C for 4 min, 95 °C for 30 s, optimal temperature of the primers for 30 s, and 65 °C for 30 s). The relative expression of the target genes was calculated using the 2^−ΔΔCt^ algorithm. Glyceraldehyde 3-phosphate dehydrogenase (GAPDH) was used as the reference gene for all target genes. Table 1 depicts the sequences of PCR primers used in this study.

### 2.10. Protein Extraction and Western Blotting

RIPA lysis buffer (MCE, HY-K1001) and 10% protease inhibitor cocktail (MCE, HY-K0010) were used for total protein extraction of the mPFC (*n* = 4 per group) and colon (*n* = 4 per group). Protein concentrations were detected using the BCA Protein Assay Kit (Promega Biotech, Beijing, China) and a microtiter plate reader (Thermo Scientific, Waltham, MA, USA). For Western blotting, the proteins were separated through sodium dodecyl sulfate-polyacrylamide gel electrophoresis and transferred onto 0.45 µm polyvinylidene difluoride membranes (Millipore, Boston, MA, USA). All membranes were incubated with primary antibodies against TPH1 (CY5574, Abways, Shanghai, China), TPH2 (AY0821, Abways, Shanghai, China), SERT (382321, Zenbio, Chengdu, China), 5-HT_1B_R (DF3497, Affinity, Golden, CO, USA), and GAPDH (ab8245, Abcam, Cambridge, MA, UK) at 4 °C overnight, followed by 1 h incubation at room temperature with secondary antibodies. The image was photographed using a Syngene GBox Imaging System (Gene Company, Shanghai, China) and an enhanced chemiluminescence solution (Millipore, Boston, MA, USA). Lastly, the expression level of the target protein bands was measured using Image Lab (version 3.0) software.

### 2.11. RNA Sequencing Analysis

The mPFCs of the PBS and LPS groups were subjected to RNA sequencing (*n* = 10 per group). The sequencing experiment was conducted using the Illumina Truseq^TM^ RNA sample prep kit, according to the manufacturer’s protocol. Total RNA was extracted from the tissue samples. The concentration and purity of the proposed RNA were examined using Nanodrop2000 (Gene Company, Shanghai, China). Then, mRNA was enriched using Oligo(dT) beads. With the addition of fragmentation buffer, mRNA broke, and small fragments of approximately 300 bp were isolated using magnetic beads. In the presence of reverse transcriptase, first-strand cDNA was synthesized using mRNA as a template, which was followed by second-strand synthesis. Then, End Repair Mix and poly (A) were added to the cDNA fragments. Finally, mRNA sequencing was performed at Majorbio Company (Shanghai, China) using the Illumina Novaseq 6000 sequencing platform (Illumina, San Diego, CA, USA). DESeq2 was used to identify differentially expressed genes (DEGs) with a fold change of >1 and *p* < 0.05. To investigate the DEG functions, Gene Ontology (GO), Kyoto Encyclopedia of Genes and Genomes (KEGG), and Reactome enrichment analyses were executed. 

### 2.12. Tryptophan Target Metabolomics

The mPFCs of the PBS (*n* = 10) and LPS (*n* = 10) offspring were sent for tryptophan target metabolomics. Each group contained two replicates. For further testing, a 50 mg aliquot of each individual sample was carefully weighed. After 500 μL of extract solution (methanol: acetonitrile: H_2_O = 2:2:1, precooled at −40 °C, containing 0.1% formic acid and an isotopically labeled internal standard mixture) was added, the samples were vortexed for 30 s, homogenized at 35 Hz for 4 min, and sonicated for 5 min in an ice-water bath. The homogenate and sonicate circles were performed twice before subsiding for 1 h at −40 ℃. Following centrifugation (15 min, 12,000 rpm, and 4 °C), a 320 μL aliquot of the supernatant was transferred to an Eppendorf tube. The supernatant was then dried under a moderate nitrogen stream before being reconstituted in 80 μL of water containing 0.1% formic acid. The clear supernatant was used for UHPLC-MS/MS analysis after centrifugation (15 min, 12,000 rpm, and 4 °C). The UHPLC separation was performed using an EXIONLC System (Sciex) outfitted with a Waters ACQUITY UPLC HSS T3 column (100 × 2.1 mm, 1.8 μm, Waters, Milford, MA, USA). For assay development, a SCIEX 6500 QTRAP+ triple quadrupole mass spectrometer (Sciex) with an IonDrive Turbo V electrospray ionization interface was used. SCIEX Analyst Work Station Software (Version 1.6.3) and Sciex MultiQuant software (Version 3.0.3) were used to collect and process data.

### 2.13. Statistical Analysis

The quantitative data were presented as mean ± standard deviation (SD). Between-group comparisons were performed using the Student’s *t*-test and Graphpad Prism software (version 9.0). *p* < 0.05 was considered statistically significant.

## 3. Results

### 3.1. Maternal LPS Treatment Resulted in ASD-like Behavioral Deficits in Offspring

**Figure 2:**
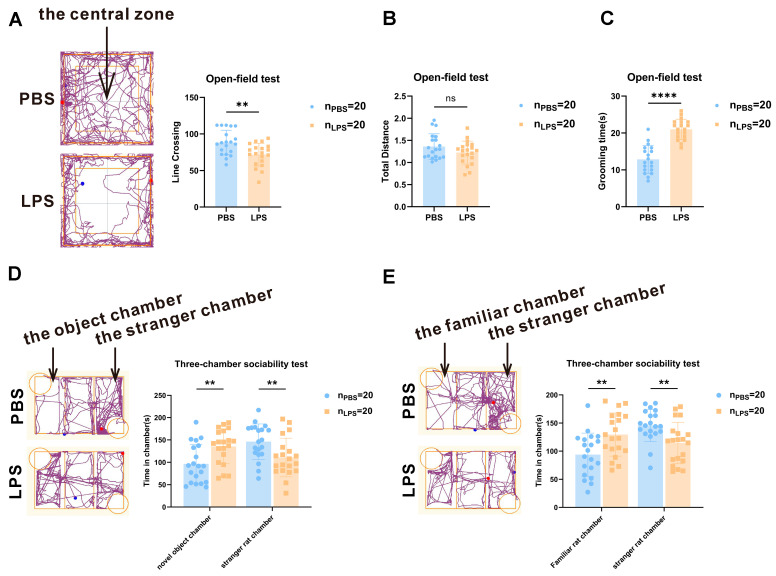
Associated behavioral test results and levels of serotonin in the mPFC of offspring rats between the LPS and PBS treatment groups during the gestational period. (**A**–**C**) Total distance traveled, times of line crossing, and grooming time in the open-field test of the two groups (*n* = 20 per group). (**D**) Rat spending time of the two groups in the novel object or stranger rat chambers during the three-chamber social test (*n* = 20 per group). (**E**) Rat spending time of the two groups in the familiar or stranger rat chambers during the three-chamber social test (*n* = 20 per group). Values are expressed as mean ± SD. Student’s *t*-test, ns, not significant, ** *p* < 0.01, **** *p* < 0.0001.

To assess if prenatal exposure to LPS causes ASD-like behaviors in offspring, we performed behavioral tests such as the open-field and three-chamber sociability tests. Using the open-field test, the autonomic inquiry activities of the rats in an unfamiliar environment were measured. After analyzing the locomotor activity, line crossing in the LPS group was found to be less than that in the PBS group (Figure 2A, *t*_38_ = 3.222, *p* < 0.05). However, no noticeable difference was observed in total traveled distance between the PBS and LPS groups (Figure 2B, *t*_38_ = 1.558, *p* > 0.05). In addition, the rats in the LPS group spent more time grooming themselves than those in the PBS group (Figure 2C, *t*_36_ = 7.674, *p* < 0.0001). These data revealed that maternal LPS exposure reduced the ability to explore novel environments but increased repetitive behaviors and did not impair the locomotor activity of offspring. 

A three-chamber sociability test can be used to evaluate the social behavior of rats and determine whether it is abnormal. Figure 2D shows that the LPS offspring spent considerably more time in the novel object chamber (*t*_38_ = 3.013, *p* < 0.01) and less time in the stranger rat chamber (*t*_38_ = 2.712, *p* < 0.01) than the PBS offspring. Additionally, Figure 2E shows that on the second day, the LPS group spent more time in the familiar rat chamber (*t*_38_ = 2.938, *p* < 0.01) and less time in the stranger rat chamber (*t*_38_ = 2.977, *p* < 0.01). These findings suggest that maternal LPS exposure impairs social interaction and social novelty recognition in its offspring.

### 3.2. Maternal LPS Treatment Might Impact Serotonin Metabolism and the Neuronal System of the Offspring mPFC

**Figure 3:**
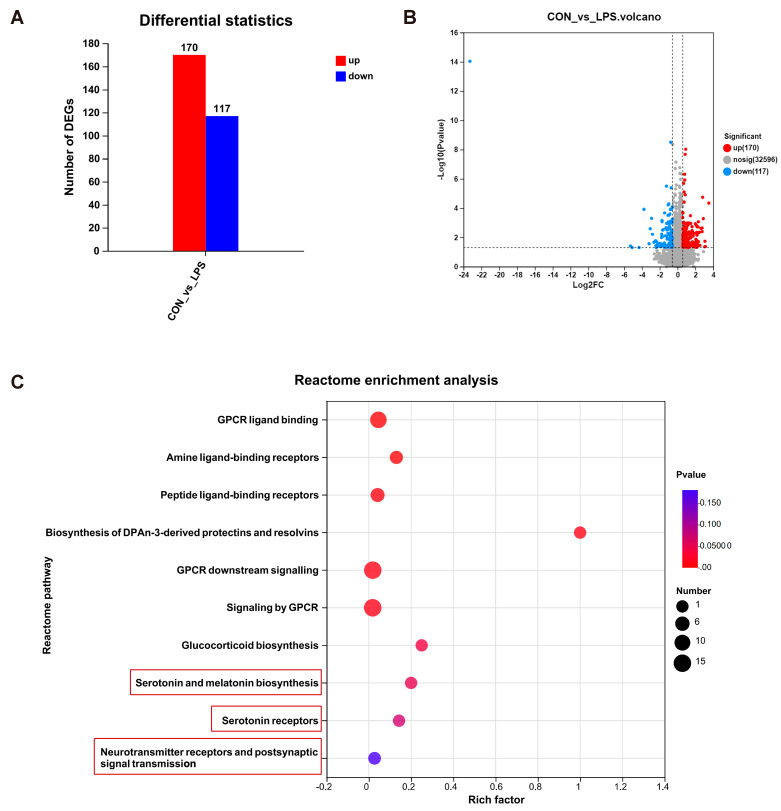
RNA-seq analysis of the offspring rat mPFC between the LPS and PBS treatment groups during the gestational period (*n* = 10 per group). (**A**) Differentially expressed gene numbers in the two different treatment groups. (**B**) Volcano plot of the differentially expressed genes (DEGs). (**C**) Representative significantly enriched Reactome pathways.

To characterize alterations in gene expression triggered by gestational LPS exposure, RNA-seq was performed using the mPFC of the PBS and LPS groups. In total, 287 DEGs were discovered. Of these DEGs, 170 were upregulated and 117 were downregulated (Figure 3A). The volcano plot is presented in Figure 3B. 

GO, KEGG, and Reactome pathway enrichment studies were conducted to gain a deeper understanding of the identified DEGs. The GO analysis and KEGG analysis suggested that DEGs were enriched in synaptic-related GO terms and tryptophan metabolism pathways (Figure S1). Reactome analysis displayed serotonin system-related enrichment pathways, including serotonin and melatonin biosynthesis and 5-HTRs (Figure 3C). In addition, the G protein-couple receptor signaling pathway, which is typically believed to be intimately connected to 5-HTRs, was also enriched. Intriguingly, we also observed that neurotransmitter receptors and postsynaptic signal transmission were enriched (Figure 3C). Collectively, maternal LPS exposure may cause changes in serotonin metabolism and the nervous system of the offspring’s mPFC.

### 3.3. Maternal LPS Treatment Increased the 5-HT Level in the mPFC of Offspring

**Figure 4:**
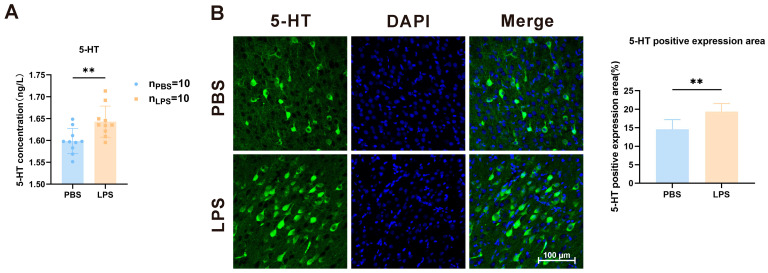
Levels of serotonin in the mPFC of offspring rats between the LPS and PBS treatment groups during the gestational period. (**A**) Concentration changes in serotonin in the mPFC of offspring rats between the LPS and PBS groups by ELISA (*n* = 10 per group). (**B**) Location and expression level of serotonin in the mPFC of offspring (*n* = 4 per group). Values are expressed as mean ± SD. Student’s *t*-test, ** *p* < 0.01.

The 5-HT content of the mPFC of 2-week-old offspring was determined using the enzyme-linked immunosorbent assay and immunofluorescence staining to explore whether LPS treatment during gestation causes any change in the serotonin system in the mPFC. As demonstrated in Figure 4A, the LPS offspring exhibited a considerable increase in 5-HT levels (*t*_18_ = 3.065, *p* < 0.001). Figure 4B displays the fluorescence expression area of 5-HT in the mPFC between the PBS and LPS offspring. According to the measured results, maternal LPS exposure significantly elevated 5-HT expression in the mPFC of the offspring (Figure 4B, *t*_10_ = 3.472, *p* < 0.01). Thus, LPS exposure during the pregnancy period induces the serotonin system in the mPFC of the LPS offspring.

### 3.4. Maternal LPS Treatment Led to Abnormal Tryptophan Metabolism in the Offspring mPFC

**Figure 5:**
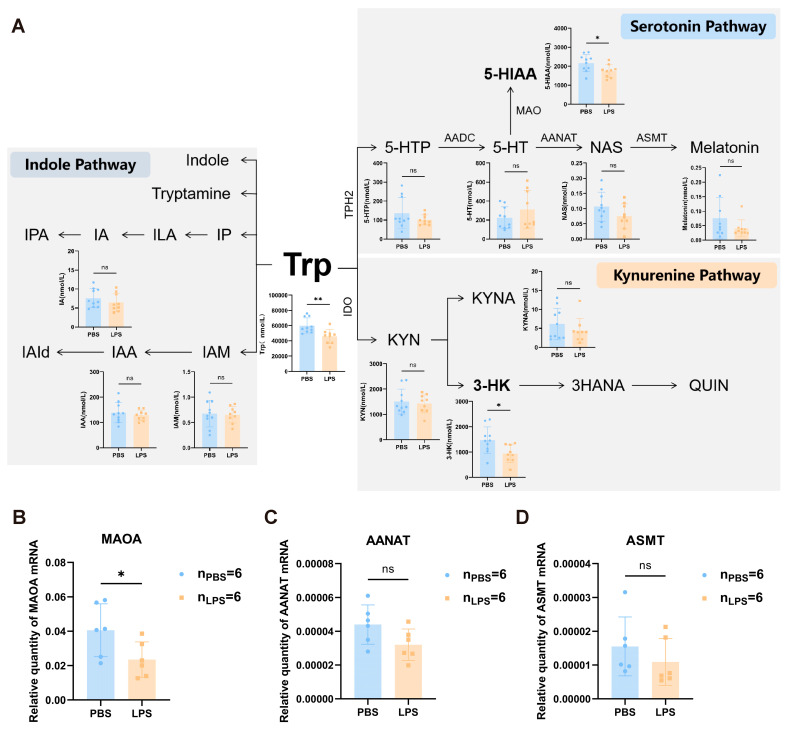
The changes in tryptophan metabolism and the expression levels of MAOA, AANAT, and ASMT in the mPFC of offspring rats between the LPS and PBS treatment groups during the gestational period. (**A**) Analysis of tryptophan target metabolomics in the prefrontal cortex of offspring rats between the LPS and PBS treatment groups during the gestational period (*n* = 10 for PBS, *n* = 9 for LPS). (**B**–**D**) The mRNA expression levels of MAOA, AANAT, and ASMT in the mPFC (*n* = 6 per group). Values are expressed as mean ± SD. Student’s *t*-test, ns, not significant, * *p* < 0.05, ** *p* < 0.01.

Tryptophan metabolism occurs through the kynurenine or serotonin pathway and produces bioactive metabolites [17]. Gut microbes may also convert tryptophan into indole and its derivatives [18]. To further to validate the changes in tryptophan metabolism following gestational LPS challenge, tryptophan target metabolomics was performed using the mPFC of 2-week-old offspring rats. The results revealed that tryptophan (Trp) levels were statistically lower in the LPS offspring than in the PBS offspring (Figure 5, *t*_17_ = 3.220, *p* < 0.01). The 5-HT level in the serotonin pathway exhibited a slightly rising trend in the LPS group, although no significant difference was observed between the LPS and PBS groups. However, the amount of 5-hydroxyindoleacetic acid (5-HIAA), a downstream 5-HT metabolite, was significantly lower in the LPS group than in the PBS group (Figure 5A, *t*_17_ = 2.327, *p* < 0.01). Furthermore, in the kynurenine pathway, 3-hydroxykynurenine (3-HK) concentration was obviously lower in the LPS group than in the PBS group (Figure 5A, *t*_17_ = 2.601, *p* < 0.05). Whereas no distinct differences in the indole pathway were observed between the LPS and PBS groups (*t*_17_ < 2, *p* > 0.05). We then detected the mRNA expression levels of 5-HT catabolic enzymes, including monoamine oxidase type A (MAOA), arylalkylamine N-acetyltransferase (AANAT), and acetylserotonin O-methyltransferase (ASMT). The findings demonstrated that the LPS-exposed offspring exhibited considerably lower MAOA mRNA expression levels (Figure 5B, *t*_10_ = 2.263, *p* < 0.05). Meanwhile, AANAT and ASMT trended toward lower mRNA expression levels (Figure 5C,D *t*_10_ < 2, *p* > 0.05). According to the aforementioned findings, LPS therapy during pregnancy may affect tryptophan metabolism, especially the kynurenine and serotonin pathways, in the mPFC of the offspring.

### 3.5. The 5-HT Receptor Expression Level Was Induced and Neuronal Morphology Changed in the Offspring mPFC after Maternal LPS Treatment

**Figure 6:**
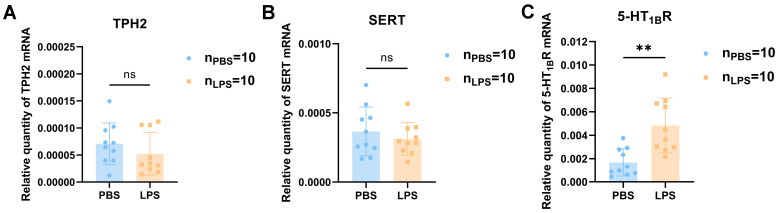

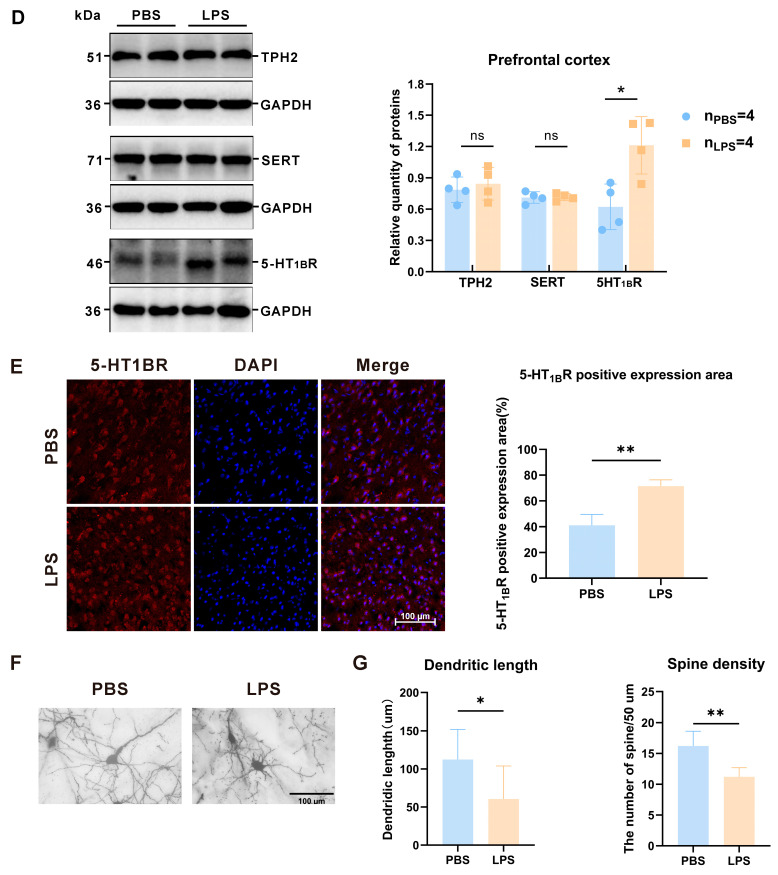
The changes in TPH2, SERT, and 5-HT_1B_R expression levels and Golgi-Cox staining in the mPFC of offspring rats between the LPS and PBS treatment groups during the gestational period. (**A**–**C**) The mRNA expression levels of TPH2, SERT, and 5-HT_1B_R in the mPFC (*n* = 10 per group). (**D**) The protein expression levels and their quantification analysis of TPH2, SERT, and 5-HT_1B_R in the mPFC (*n* = 4 per group). (**E**) Location and expression level of 5-HT_1B_R in the mPFC of offspring rats between the LPS and PBS groups by immunofluorescence staining (*n* = 4 per group). (**F**) Photomicrograph showing representative Golgi-Cox impregnation of the two groups (×400) (*n* = 4 per group). (**G**) The dendritic length and the number of spines /50µm in the LPS and PBS. Values are expressed as mean ± SD. Student’s *t*-test, ns, not significant, * *p* < 0.05, ** *p* < 0.01.

Tryptophan hydroxylase (TPH) is the rate-limiting enzyme of the 5-HT synthesis pathway, including TPH1 and TPH2, which are responsible for 5-HT synthesis of the intestinal and central nervous systems, respectively [19,20]. 5-HT operates through a group of serotonin receptors (5-HTRs) [21]. Among them, 5-HT_1B_R has been linked to social and communicative activities [22,23]. Then, the selective serotonin reuptake transporter (SERT) transports 5-HT into cells for degradation to maintain the 5-HT system’s homeostasis [24]. To determine the key points of serotonin synthesis, serotonin transporter, and 5-HTRs affected by maternal LPS treatment, changes of TPH2, SERT, and serotonin 1B receptor (5-HT_1B_R) protein expression in the mPFC of the offspring were detected by Western blotting and immunofluorescence. According to the quantitative analysis of qPCR and Western blot, the mRNA and protein expression levels of TPH2 and SERT exhibited no significant differences between the LPS and PBS groups (Figure 6A,B,D, *t*_6_ < 2, *p* > 0.05; *t*_6_ < 2, *p* > 0.05). The 5-HT_1B_R mRNA and protein expression levels were significantly increased in the LPS offspring compared with the PBS offspring (Figure 6C,D, *t*_6_ = 3.357, *p <* 0.05). Using immunofluorescence staining, we observed that the 5-HT_1B_R levels were noticeably higher in the LPS group than in the PBS group (Figure 6E, *t*_4_ = 5.386, *p* < 0.01), which is consistent with the qPCR and Western blot results. These results reveal that maternal LPS therapy affects serotonin biological function through the expression of its receptor 5-HT_1B_R in the mPFC, whereas it does not impact 5-HT synthesis and reabsorption in the mPFC. Neurons were morphologically observed using Golgi-Cox staining in the mPFC of 2-week-old offspring to further investigate the association between the change in the 5-HT level and ASD-like behavior resulting from maternal LPS exposure. The dendritic branches of the neurons were noticeably shorter in the LPS group than in the PBS group (Figure 6F). According to the quantitative analysis results, dendrite length of the LPS group was significantly lower than that in the PBS group (Figure 6G, *t*_14_ = 2.470, *p* < 0.05). Meanwhile, a remarkable reduction in spine density was observed in the LPS offspring (Figure 6G, *t*_8_ = 3.978, *p* < 0.01). Together, the present findings confirm that maternal LPS exposure contributes to the abnormal morphology of dendrites during the early postnatal period. 

### 3.6. 5-HT Synthesis Was Induced and Reabsorption Was Reduced in the Offspring Colon after Maternal LPS Treatment

**Figure 7:**
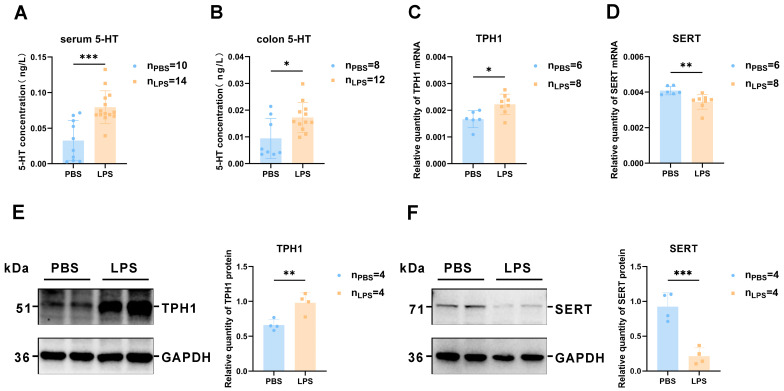
The levels of serotonin and changes in TPH1 and SERT expression levels in the serum and colon of offspring rats between the LPS and PBS treatment groups during the gestational period. (**A**) Concentration changes in serotonin in the serum of offspring rats between the LPS and PBS groups by ELISA (*n* = 10 for PBS, *n* = 14 for LPS). (**B**) Concentration changes in serotonin in the colon of offspring rats between the LPS and PBS groups by ELISA (*n* = 10 for PBS, *n* = 14 for LPS). (**C**,**D**) The mRNA expression levels of TPH1 and SERT in the mPFC (*n* = 6 for PBS, *n* = 8 for LPS). (**E**,**F**) The protein expression levels and their quantification analysis of TPH1 and SERT in the colon (*n* = 4 per group). Values are expressed as mean ± SD. Student’s *t*-test, * *p* < 0.05, ** *p* < 0.01, *** *p* < 0.001.

Considering that 90% of 5-HT is synthesized in the intestine, 5-HT levels as well as TPH1 and SERT protein expression were assessed using Western blotting and immunofluorescence. Figure 7A demonstrates that LPS offspring have higher serum 5-HT levels than the PBS group, consistent with the alteration in the mPFC (*t*_22_ = 4.463, *p* < 0.001). Figure 7B reveals that the 5-HT content of colon was also higher following maternal LPS stimulation (*t*_18_ = 2.680, *p* < 0.05), also consistent with the alteration in the mPFC. Furthermore, the mRNA and protein expression levels of both TPH1 and SERT displayed notable elevation and reduction, respectively, in the colon of LPS offspring at 2 weeks compared with the PBS offspring (Figure 7C–F, *t*_22_ = 2.912, *p* < 0.05; *t*_22_ = 3.293, *p* < 0.01; *t*_6_ = 3.898, *p* < 0.01; *t*_6_ = 6.134, *p* < 0.001). To sum up, these are crucial findings related to the understanding of the increased 5-HT level in the mPFC, and we speculated that the changes in 5-HT metabolism in the colon may have a certain effect on the 5-HT level in the mPFC during the period of early-life neurodevelopment.

## 4. Discussion

Immune system activation during pregnancy increases the risk of neurodevelopmental disorders in the offspring. The method of constructing ASD animal models through maternal LPS exposure is well established [25,26]. Considering that the 2-week-old rats were not yet weaned and were too young for behavioral experiments, we performed behavioral tests on 6- to 8-week-old rats. In the present study, symptoms analogous to those associated with ASD in adult LPS offspring were observed, such as repetitive stereotype behaviors, impaired social interaction, and social novelty recognition. These reveal that the ASD-like offspring model was successfully established through the maternal LPS challenge.

The association between ASD and 5-HT system dysfunction has been known for a long time [27,28]. Previous investigations in ASD patients revealed a reduction in tryptophan metabolism, which is the only source of the neurotransmitter serotonin [29]. Furthermore, Tanaka M. revealed that dietary tryptophan restriction can normalize ASD-relevant social deficits [30]. To clarify whether alterations in tryptophan metabolism also occurred in our model, we conducted RNA-seq, in which DEGs were enriched in the tryptophan metabolism pathway, serotonin and melatonin biosynthesis, and the 5-HTR pathway. It has been reported that approximately 30% of those with ASD had elevated 5-HT levels in their whole blood [31,32,33]. Previous articles published by our research group show that the severity of autistic symptoms is closely related to serum 5-HT levels [13]. Our recent study also found metabolic disturbances in ASD children involved in 5-HT-related pathways [14]. In addition, elevations or reductions in 5-HT levels in multiple brain regions have been demonstrated in various ASD animal models except for the LPS-induced ASD model [10,11,12]. Here, a higher 5-HT level was recorded in the mPFC of the ASD offspring than in the PBS offspring. Then, tryptophan target metabolomics in the mPFC revealed the decrease in Trp and 5-HIAA. Moreover, the 5-HT concentration in the LPS-exposed offspring displayed a slightly rising trend, albeit nonsignificant. Serotonin degradation primarily occurs via the conversion of MAOA to the major serotonin metabolite, 5HIAA. Importantly, serotonin otherwise serves as an intermediate substrate for melatonin synthesis, which occurs via AANAT and ASMT [7,34,35]. Thus, the 5-HT level is influenced by synthetic enzymes as well as catabolic enzymes. mRNA expression levels detected in our study exhibited a notable reduction in MAOA. Bortolato M et al. found that MAOA KO mice displayed neuropathological alterations reminiscent of typical ASD feature [36]. Similarly, early exposure of rats to MAOA inhibitor has been shown to produce constant repetitive stereotypic behaviors [37]. The downtrend of AANAT and ASMT were also found in our study, thereby accounting for the rising trend of 5-HT levels. These data indicate that ASD characteristics were preceded by changes in the 5-HT system.

Many key proteins are involved in the anabolism, catabolism, and function of 5-HT. Most reports have supported the conclusion that TPH2, the initial and rate-limiting enzyme in 5HT production, is related to ASD [38,39]. For example, *Tph2*^−/−^ mice have ASD-related behavioral consequences [40,41]. SERTs are located on presynaptic serotonin nerve terminals as well as on axons and serotonin cell bodies in the raphe [42]. By serving as transporter molecules, SERTs can effectively manage serotonin concentrations by rapidly clearing serotonin from the synaptic gap [43]. A previous study revealed that the 5-HT level is increased in autistic children, and SERT is also increased to clean excess 5-HT from the synaptic cleft, thus limiting its action [44]. At the same time, Miho Tanaka noted that *Sert*^−/−^ mice also exhibited ASD-associated behavioral changes [30]. However, contrary to expectations, notable differences were observed neither in TPH2 nor in SERT between the LPS and PBS groups. This may be because we chose 2 weeks of age as the test point, when the offspring are still juveniles, whereas other experiments used adult rats. Moreover, different models may have different results. 5-HT can play its physiological function only when it binds to the receptor, and different receptors have different physiological functions [45]. Among them, rodent social and communicative activities have been linked to 5-HT_1B_R [22,23]. Lawson SK recently revealed that the 5-HT_1B_R agonist challenge induces autism-related behavior in mice [46]. According to these studies, maternal LPS stimulation increased both mRNA and protein expression levels of 5-HT_1B_R in the mPFC of 2-week-old offspring. These results imply that LPS had no effect on 5-HT synthesis or degradation but did affect the expression of the receptor in the mPFC during the early neurodevelopment period.

Numerous studies have elucidated that the serotonin system is involved in the growth of neuronal systems [3,47]. Besides, 5-HT regulates multiple aspects of neural development, including neuronal differentiation and migration, synaptic plasticity, axonal outgrowth, and control of dendritic spine shape and density [7,8,48]. Most investigations have revealed that maternal LPS exposure reduces the number of dendritic spines and shortens dendritic length in the brain tissue of offspring, despite some contrary findings [40,49]. Our findings are in line with those of Moogeh’s study [49], as a significant reduction in dendritic length and spine density was observed following LPS exposure during pregnancy. According to these findings, maternal LPS exposure damaged the dendritic structure, and morphological abnormalities might be caused by alterations in the 5-HT level.

5-HT cannot cross the BBB. Nonetheless, the BBB does not fully mature until tight junction-specific proteins appear in rats at approximately postnatal day 20 [50]. Moreover, high 5-HT expression in the colon has been documented in ASD animal models [51]. We therefore speculated that 5-HT in the colon may penetrate the brain at 2 weeks of age, when the BBB is not mature. Consistent with our speculation, the 5-HT concentration in the serum and colon of LPS offspring was indeed elevated. Furthermore, our results are similar to those of Xiao, L [52], as an increase in TPH1 (a rate-limiting enzyme for peripheral 5-HT synthesis) and SERT mRNA and protein expression levels of colon was detected in our work. These findings may lead to a better understanding of the increase in 5-HT levels in the mPFC of the offspring. 

Although our study made some important discoveries, there are limitations. First, multiple comparisons were not conducted in the current study. Second, a major experiment is urgently required to support the colon-central nervous system crosstalk to regulate 5-HT metabolism. Finally, further research is also required to fully comprehend the mechanisms through which the 5-HT system regulates nervous system development. 

## 5. Conclusions

Overall, our work clarifies that maternal LPS exposure increases the 5-HT level in the mPFC of the young offspring, which may be attributable to the ASD-related behavior in adult rats that is potentially induced by the high 5-HT level in the colon.

## Figures and Tables

**Table 1:** Sequences of the specific primers used for rat genes.

Name	Sequence
TPH1	CCATCTTCCGAGAGCTAAACAAA
	TCTTCCCGATAGCCACAGTATT
TPH2	GTGACCCTGAATCCGCCTG
	GGTGCCGTACATGAGGACT
SERT	GGCGGAGATGAGGAATGAAGATGTG
	TGGATGCTGGCATGTTGGCTATTG
5-HT_1B_R	CACCCTTCTTCTGGCGTCAAGC
	CCGTGGAGTAGACCGTGTAGAGG
MAOA	GACACGCTCAGGAATGGGACAAG
	ACAGGAACCACAGGGCAGATACC
AANAT	GGTTCACTTTGGGACAAGGAGAGAC
	GAAGGTATCGCCACAGCAGGAC
ASMT	AGGGAGAGACGTTGGAATCAGAGG
	CTTGCTTGAGGGTGCCACTTCTG
GAPDH	ACGGCAAGTTCAACGGCACAG
	CGACATACTCAGCACCAGCATCAC

## Data Availability

The raw data supporting the conclusions of this article will be made available by the authors, without undue reservation.

## Supplementary Materials

The following supporting information can be downloaded at: https://www.mdpi.com/article/10.3390/brainsci13060958/s1, Figure S1: RNA-seq analysis of the offspring rat mPFC between the LPS and PBS two treatment groups during gestational period (n = 10 per group). (**A**) Representative significantly enriched GO pathways. (**B**) Representative significantly enriched KEGG pathways.
