# Supplementary material for: Maternal LPS Exposure Enhances the 5-HT Level in the Prefrontal Cortex of Autism-like Young Offspring
Source: Brain Sci. 2023 Jun 15;13(6):958. doi: 10.3390/brainsci13060958 (PMC10295954; doi:10.3390/brainsci13060958)
Supplement: Supplementary file 1 [file brainsci-13-00958-s001.zip › brainsci-2390260-supplementary.pdf]

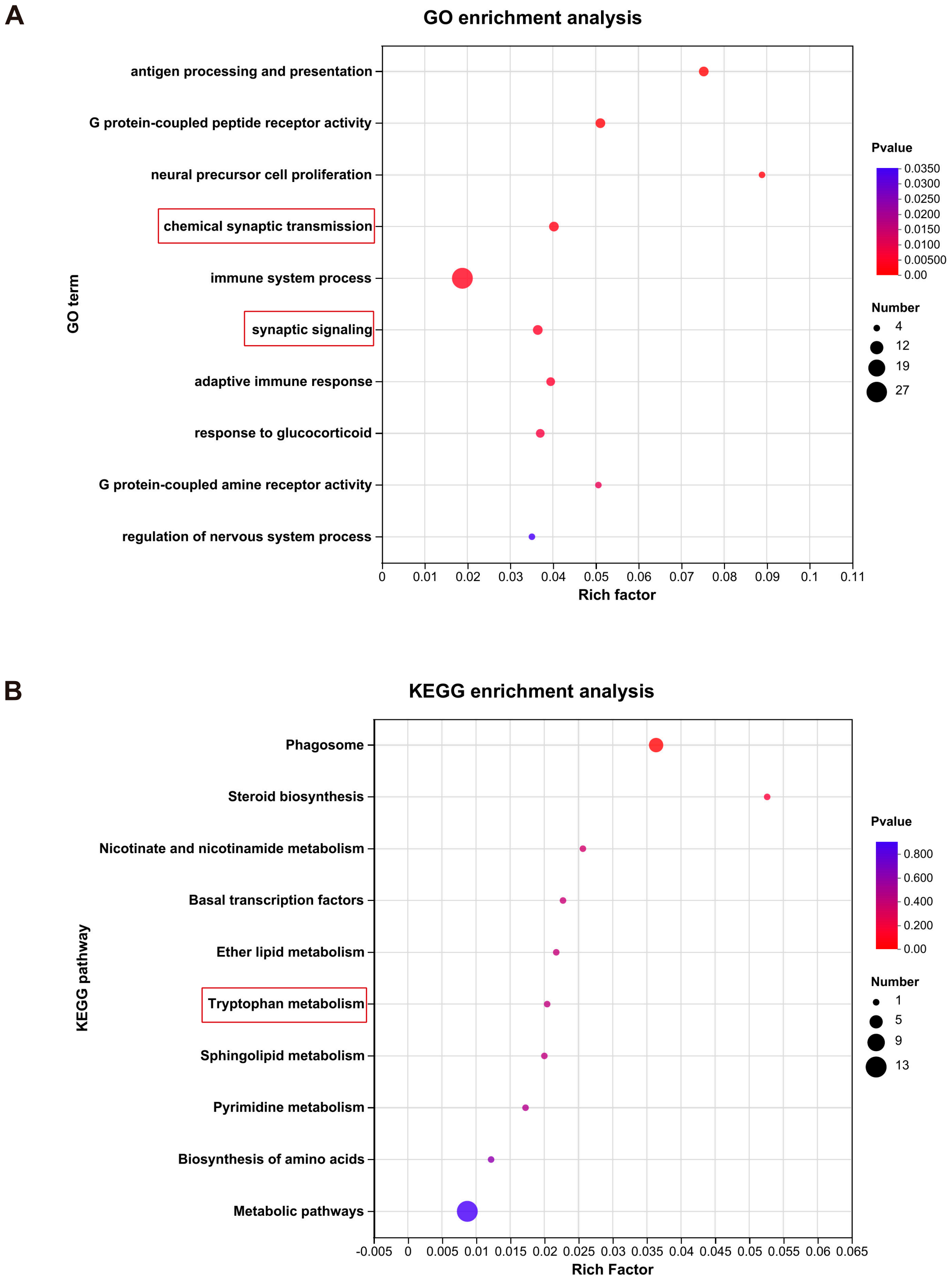

Figure S1. RNA-seq analysis of the offspring rat mPFC between the LPS and PBS two treatment groups during gestational period (n=10 per group). **(A)** Representative significantly enriched GO pathways. **(B)** Representative significantly enriched KEGG pathways.
